# Cwp2 from *Clostridium difficile* exhibits an extended three domain fold and cell adhesion *in vitro*

**DOI:** 10.1111/febs.14157

**Published:** 2017-07-23

**Authors:** William J. Bradshaw, Jonathan M. Kirby, April K. Roberts, Clifford C. Shone, K. Ravi Acharya

**Affiliations:** ^1^ Department of Biology and Biochemistry University of Bath UK; ^2^ Public Health England Salisbury UK

**Keywords:** bacterial adhesion, cell wall, *Clostridium difficile*, colitis, crystal structure

## Abstract

Colonization of the gut by *Clostridium difficile* requires the adhesion of the bacterium to host cells. A range of cell surface located factors have been linked to adhesion including the S‐layer protein LMW SLP and the related protein Cwp66. As well as these proteins, the S‐layer of *C. difficile* may contain many others. One such protein is Cwp2. Here, we demonstrate the production of a *C. difficile* strain 630 *cwp2* knockout mutant and assess the effect on the bacterium. The mutant results in increased TcdA (toxin A) release and impaired cellular adherence *in vitro*. We also present the extended three domain structure of the ‘functional’ region of Cwp2, consisting of residues 29–318 at 1.9 Å, which is compared to that of LMW SLP and Cwp8. The adhesive properties of Cwp2 and LMW SLP, which are likely to be shared by Cwp8, are predicted to be mediated by the variable loop regions in domain 2.

**Databases:**

Structural data are available in the PDB under the accession number 5NJL.

**Abbreviations**
CWBD: cell wall‐binding domain
Cwp: cell wall protein
LMW SLP: low molecular weight S‐layer protein

## Introduction

*Clostridium difficile* is the primary causative agent of antibiotic associated diarrhoea. Increasing resistance to antibiotics in recent decades has resulted in a reduction in the efficacy of standard methods of treatment 1, 2. This presents a clear need for a greater understanding of the bacterium, so that alternative methods of treating *C. difficile* infection (CDI) may be developed.

*Clostridium difficile* has a number of virulence factors. The pathogenic strains of *C. difficile* produce two potent exotoxins, TcdA and TcdB (also known as Toxin A and Toxin B), which induce mucosal inflammation and diarrhoea 3, 4. Another important factor that has been identified is the paracrystalline protein array on the surface of *C. difficile* cells known as an S‐layer. S‐layers have been shown to be involved in a range of important cellular functions including host cell adhesion and immune system evasion 5. The S‐layer of *C. difficile* is primarily formed of the low‐ and high‐molecular‐weight S‐layer proteins (LMW SLP and HMW SLP, respectively), coded for by the gene *slpA*, but may contain up to 28 SlpA paralogues 6. HMW SLP possesses three cell wall‐binding 2 (CWB2) domains which mediate attachment to the cell wall through interaction with the surface exposed polysaccharide PSII 7. These domains are shared by the other proteins associated with the S‐layer, while many also possess a ‘functional’ region. One such protein is Cwp2, the gene for which was first identified in 2001 as part of a cluster of S‐layer‐associated genes surrounding *slpA* 8, 9.

*cwp2*, which is found within the *slpA* locus and is transcribed during normal growth 8, was initially claimed to be at the start of a polycistronic operon also encoding an LmbE‐like deacetylase and Cwp66 10. The presence of a terminator between *cwp2* and the deacetylase gene, however, suggests this is not the case 11. Cwp66 has been shown to have adhesive properties 12 and the possibility that the three proteins may work together to form an adhesin complex has been suggested 10, although, to date, no such complex has been found on the cell surface. A comparison of 57 isolates indicated that *cwp2* shows significant variation exceeded within the *slpA* locus only by the surrounding *slpA*,* cwp66* and *secA2* genes 13. The majority of *cwp* genes have been shown to be upregulated in sub‐MIC ampicillin, clindamycin and metronidazole, while only *cwp2*,* cwp84*,* cwp6* and *cwp7* are upregulated in the presence of amoxicillin 14.

Cwp2 was initially identified in all guanidine cell surface extracts from strains covering a range of serotypes and ribotypes of *C. difficile*. The protein was reported to cross‐react with all antisera raised against five different strains 15. Proteomic analysis of low pH or lysozyme cell wall extracts confirmed that the 66 kDa protein found in surface extracts was Cwp2 16 and the protein has also been shown to be present in the *C. difficile* spore coat 17. An N‐terminal 38–41 kDa protein fragment of Cwp2 has been shown to be present in culture supernatant, particularly during conditions promoting high toxin production 18.

Cwp2 appears to be highly immunogenic as virtually all patients produce antibodies that recognize the protein 19. This, coupled with a lack of Cwp2 in some strains 20, indicates that Cwp2 is not necessary for CDI and that antibodies to the protein are unlikely to be protective against CDI. However, the altered S‐layer caused by the lack of Cwp2 in strain 167, may be negated by changes in other virulence‐associated factors such as increased adhesion, increased toxin production or the presence of a glycosylation gene cluster 13. Notably, a Cwp84 mutant (with an immature S‐layer *in vitro*) is still able to cause CDI in the hamster model, this may be due to the action of host proteases, but could potentially be directly overcome by the bacterium through some unknown means 21.

Until recently, the only structures of proteins associated with the S‐layer that had been determined were of truncated LMW SLP 22 and the cysteine protease and ‘lectin‐like’ domains of Cwp84 23. However, the structures of full length Cwp6 and Cwp8, which include the cell wall‐binding domains, were recently reported, shedding light on the mechanism of binding to PSII 24. As well as this, the previously uncharacterized N‐terminal functional region of Cwp8 was shown to be elongated and composed of three domains.

Here, we present a truncated structure of the functional region (residues 29–318) of Cwp2, which shows a similar fold to that of Cwp8. We also demonstrate the production of a *C. difficile cwp2* knockout mutant, which shows no impairment to growth, spore formation or ability to cause CDI in the hamster model. The mutation did, however, result in an increase in *in vitro* TcdA release into culture medium, suggesting that the bacterium may be stressed. The mutant also showed an impaired ability to adhere to Caco‐2 cells, indicating a potential role for Cwp2 as an adhesin.

## Results

### The structure of Cwp2

**Figure 1:**
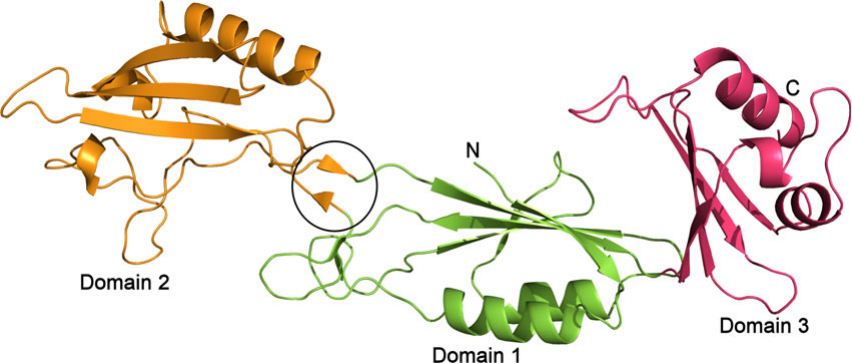
The structure of Cwp2 – Domain 1 is shown in green, domain 2 in orange and domain 3 in pink. The functional region of Cwp2 assumes an extended three domain structure. Domain 1 possesses a two‐layer sandwich fold. Domain 2 has a single α‐helix, a small β‐sheet and significant loop regions. Domain 2 is connected to domain 3 via a strand in domain 1. Domain 3 forms a similar two‐layer sandwich fold to domain 1. The hinge region, which allows domain 2 to rotate, is circled.

The structure of the functional region of Cwp2, consisting of residues 29–318 (two residues at the N‐terminus and four residues at the C‐terminus are not visible) has been determined to 1.9 Å (Fig. 1). The protein was crystallized in the hexagonal space group P6_3_22 with one protein chain, 20 sulphate ions and 229 water molecules in the asymmetric unit. The details of crystallographic statistics are given in Table 1. Coordinates have been deposited with the protein data bank with accession code 5NJL. All sulphate ions appear loosely bound on the periphery of the protein and are likely to be present as a result of the crystallization conditions, as such, they are not believed to be functionally relevant.

**Table 1:** Crystallographic and refinement statistics. Inner and outer shell statistics are given in square and round brackets respectively

Crystallographic statistics	
Space group	P6_3_22
Cell dimensions (Å, °)	134.8, 134.8, 102.8, 90.0, 90.0, 120.0
Resolution (Å)	[120.83–9.11] (1.94–1.90)
*R*_merge_	[0.051] 0.166 (4.497)
*R*_meas_	[0.052] 0.169 (4.624)
*R*_pim_	[0.008] 0.021 (0.772)
CC_1/2_	[1.000] 1.000 (0.685)
Mean <I/σI>	[74.7] 21.6 (1.5)
Completeness (%)	[100.0] 100.0 (100.0)
Total reflections	[29 049] 2 741 248 (97 683)
Total unique reflections	[495] 43 871 (2766)
Multiplicity	[58.7] 62.5 (35.3)
Refinement statistics	
*R*_work_/*R*_free_	0.215/0.245
RMSDs	
Bond Lengths (Å)	0.010
Bond Angles (°)	1.440
Ramachandran statistics (%)	
Favoured	97.2
Allowed	2.4
Outliers	0.4
Average B‐factors	
Protein	41.6
Sulphate ion	92.5
Water	40.3
Number of atoms	
Protein	2238
Sulphate ion	100
Water	229
PDB Code	5NJL

**Figure 2:**
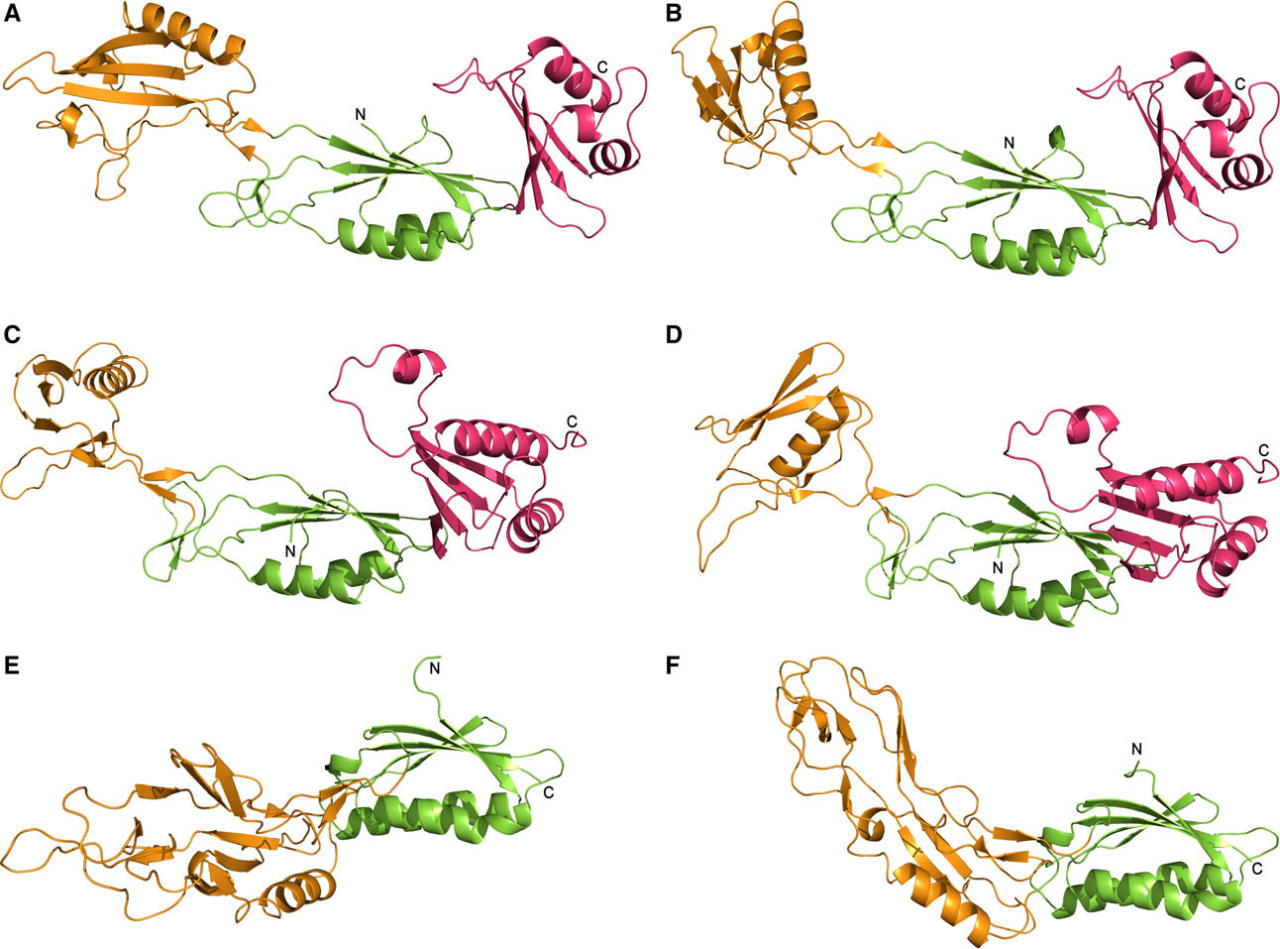
Comparison between Cwp2, Cwp8 and LMW SLP and flexibility analysis – (A, C, E) The structures of Cwp2, Cwp8 (5J6Q) and LMW SLP (3CVZ), respectively. As with Fig. 1, domain 1 is shown in green, domain 2 in orange and domain 3 in pink. The flexibility of these structures and the hinge region between domains 1 and 2 was analysed with FIRST and FRODA. (B) Cwp2 after rotation of domain 2 around the hinge region. The domain is able to assume an orientation closer to that seen in Cwp8. (D) Cwp8 after rotation of domain 2 around the hinge region. The domain is able to assume an orientation closer to that seen in Cwp2. (F) LMW SLP after rotation of domain 2 around the hinge region. Although a considerably higher degree of difference is seen between this domain and domain 2 of Cwp2 and Cwp8 than between the latter two, the domain is still able to rotate and assume a position somewhat closer to that seen in Cwp2 and Cwp8.

Cwp2 possesses a similar extended fold to that of the recently reported structure of Cwp8 24, to which it has 27% amino acid identity and 44% similarity, domain 2 shows a greater level of sequence variation between the two proteins 25. Overall, domains 1 and 3 possess two‐layer sandwich folds consisting of a four‐stranded mixed β‐sheet and two α‐helices while domain 2 has a smaller antiparallel β‐sheet, one α‐helix and a large loop region. The two structures superpose with an RMSD of 3.47 Å (all atom alignment, 924 atoms – RMSDs for individual domains are given in Table 2). Domains 1 and 3 show high similarity to the equivalent domains in Cwp8 and lower similarity to each other. In the case of domain 2, while the β‐sheet and α‐helix are conserved, the loop region shows significant differences. The two domains superpose with an RMSD of 3.68 Å (261 atoms). With domains 1 and 3 superposed, domain 2 of Cwp2 is rotated approximately 40° relative to domain 2 of Cwp8. A detailed analysis of the flexibility of the domains demonstrated an ability of the interface between domains 1 and 2 to act as a hinge, so that the structures of Cwp2 and Cwp8 are able to superpose upon each other more closely (Fig. 2).

**Table 2:** Domain RMSDs (Å) between Cwp2, Cwp8 and LMW SLP. All RMSDs are for sequence‐independent alignments using all atoms, the number of atoms used is given in brackets

		Cwp8		Cwp2	
		Domain 1	Domain 3	Domain 1	Domain 3
LMW SLP	Domain 1	1.79 (263)	N/A	2.32 (271)	3.57 (96)
Cwp2	Domain 3	3.62 (98)	1.31 (276)	N/A	
	Domain 1	1.03 (309)	8.36 (237)		
Cwp8	Domain 3	7.14 (290)			

As well as similarity to Cwp8, domains 1 and 3 of Cwp2 also show significant similarity to domain 1 of LMW SLP 22. A DALI search performed with the individual domains of Cwp2 showed that all three domains bear very little similarity to any known structures other than Cwp8 and LMW SLP.

### Knockout analysis

#### Confirmation of mutation

**Figure 3:**
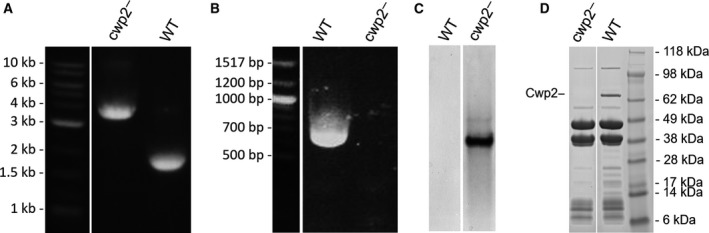
cwp2^−^ mutant characterization gels‐ (A) Agarose gel of PCR products confirming intron integration into *cwp2* using extracted gDNA (B) Agarose gel of RT‐PCR products confirming the absence of cwp2 transcript from extracted mRNA (C) Southern Blot confirming single intron insertion in *cwp2* mutant. (D) SDS/PAGE of low pH SLP extract demonstrating loss of Cwp2 from cell surface. Images have been rotated and several lanes omitted.

To analyse the role of Cwp2, the ClosTron system was used to inactivate the *cwp2* gene in *C. difficile* strain 630ΔErm forming a cwp2^−^ mutant. Insertion of the group II intron into the target gene was verified by *cwp2* gene‐specific primers (Table 3 and Fig. 3A). RT‐PCR was performed on extracted RNA using primers flanking the intron insertion sites to confirm truncation of *cwp2* mRNA upon intron insertion – no expression products were obtained for the cwp2^−^ mutant (Fig. 3B). Southern blot analysis confirmed the single chromosomal insertion of the intron. (Fig. 3C).

**Table 3:** Primers used in this study

Gene‐intron‐gene	GCTGATGTTGATAAAGATAGAAAAGTTCAA – intron – AGAGTTGAAGGAGAA
IBS	AAAAAAGCTTATAATTATCCTTAAGAAACGTTCAAGTGCGCCCAGATAGGGTG
IBS1d	CAGATTGTACAAATGTGGTGATAACAGATAAGTCGTTCAAAGTAACTTACCTTTCTTTGT
EBS2	TGAACGCAAGTTTCTAATTTCGATTTTTCTTCGATAGAGGAAAGTGTCT
CspFdx‐F1	GATGTAGATAGGATAATAGAATCCATAGAAAATATAGG
pMTL007‐R1	AGGGTATCCCCAGTTAGTGTTAAGTCTTGG
ErmRAM forward	ACGCGTTATATTGATAAAAATAATAATAGTGGG
ErmRAM reverse	ACGCGTGCGACTCATAGAATTATTTCCTCCCG
*cwp2* forward	ATGAATAAAAAAAATCTTTCTG
*cwp2* reverse	TTACCAACCTAGTATTTTAGC
*cwp2* pre intron 1000	ACTGCAGTGGCTATAGCAAA
*cwp2* post intron 1703	CCTGCTGCTAATGCATCTAC
pMTL007_F1	TGCGCCCAGATAGGGTGTTAAGT
pMTL007‐R	AGGGTATCCCCAGTTAGTGTTAAGTCTTGG

#### Analysis of surface layer proteins of surface protein mutants

In order to determine if the knockout had an effect on the surface of the bacterium, the surface proteins were removed by low pH extraction and examined by SDS/PAGE (Fig. 3D). S‐layer extracts from the Cwp2 knockout strain appeared largely similar to the wild‐type. The main S‐layer bands appeared unaffected and were present at 40 kDa and 47 kDa respectively. The knockout extract lacked a 66 kDa species. Mass spectrometric analysis of this protein in the wild‐type identified it as Cwp2, confirming the loss of the Cwp2 from the cell surface, corresponding with inactivation of *cwp2*.

#### Growth

The knockout strain appeared to have normal colony morphology and mutant cells were indistinguishable from the wild‐type under Gram stain. The growth rate of the mutant in liquid medium was determined to be 95% ± 6.2 (SEM) of the wild‐type.

#### Sporulation

cwp2^−^ cells appeared to retain their ability to sporulate with no difference to the wild‐type, (6.65 ± 0.02 log_10_ CFU·mL^−1^ WT, 6.42 ± 0.04 log_10_ CFU·mL^−1^ cwp2^−^), suggesting that the ability to form viable, heat resistant, spores was unaffected.

#### Toxin production

TcdA and TcdB are typically produced when cultures reach stationary phase – after approximately 24 h for *C. difficile* 630, and when cultures are stressed 26. The ability of cwp2^−^ mutants to produce toxin was assessed by ELISA and compared to toxin production of the wild‐type under similar growth conditions.

**Figure 4:**
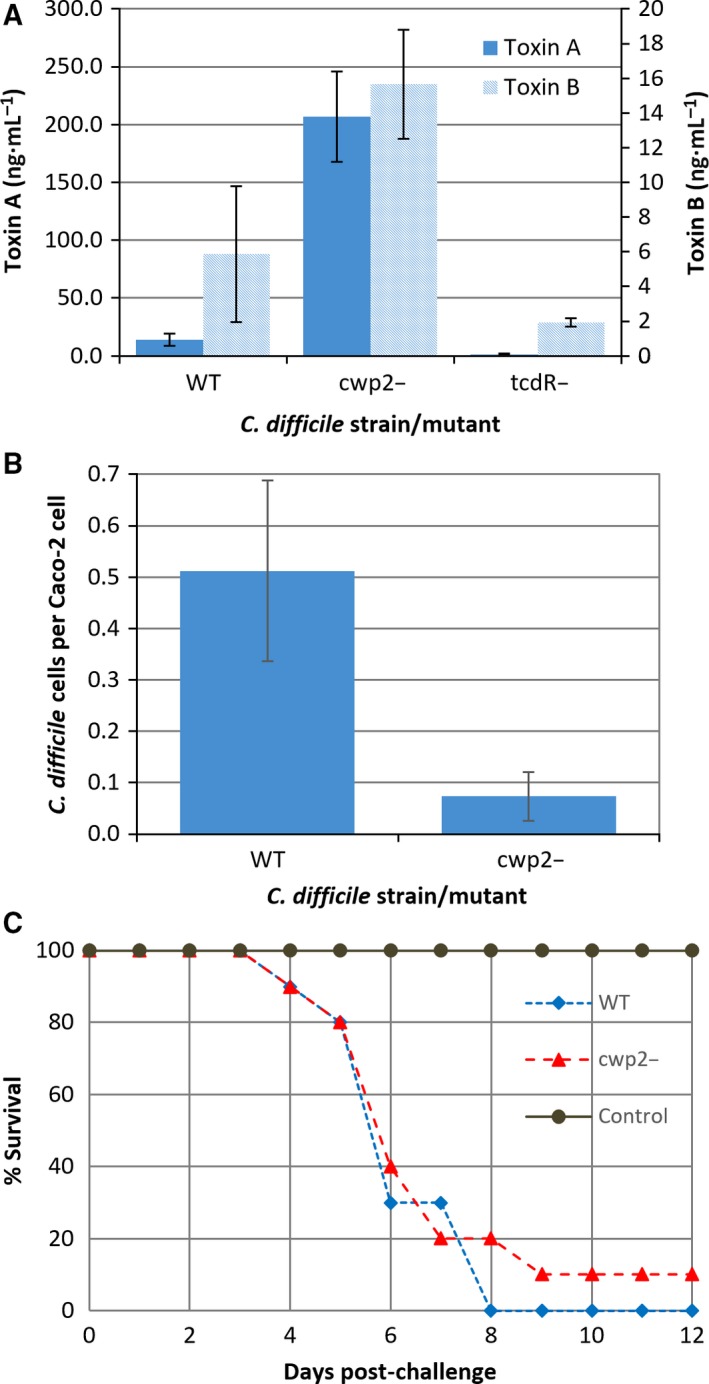
cwp2^−^ mutant characterization (A) Estimation of TcdA and TcdB concentration in culture supernatant as measured by ELISA. Strains grown in sBHI culture medium for 24 h. Limit of quantification 5 ng·mL^−1^. Data analysed with student's *t*‐test. TcdA showed a significant increase in toxin production (WT = 14.2 ± 5.2 ng·mL^−1^ (SEM), *n* = 5, cwp2^−^ = 207.0 ± 39.1 ng·mL^−1^, *n* = 5, *P* < 0.05). TcdB did not show a significant increase (WT = 5.9 ± 3.9 ng·mL^−1^, *n* = 5, cwp2^−^ = 15.7 ± 3.5 ng·mL^−1^, *n* = 4, *P* > 0.05). (B) Adherence of cwp2^−^ to Caco‐2 cells relative to 630ΔErm. Mean adhesion of the mutant (0.073 ± 0.047 bacteria per cell) was significantly lower than the wild‐type (0.51 ± 0.18) (*P* < 0.05, Student's *t*‐test). (C) Kaplan‐meier survival curve of *Clostridium difficile* 630ΔErm and cwp2^−^ in hamsters. Data show time from challenge to severe disease/death in the hamster model for CDI. *n* = 10 for test groups, *n* = 4 for control.

Wild‐type cultures produced 14.2 ± 5.2 ng·mL^−1^ (SEM) TcdA and 5.9 ± 3.9 ng·mL^−1^ TcdB. The cwp2^−^ cultures produced 207.0 ± 39.1 ng·mL^−1^ TcdA and 15.7 ± 3.5 ng·mL^−1^ TcdB (Fig. 4A). This represents a statistically significant 14‐fold average increase in TcdA production in the cwp2^−^ mutants compared to the wild‐type (*P* < 0.05, Student's *t*‐test) as measured by ELISA. The TcdB level of mutant 24 h culture supernatants demonstrated an increase, but it was not significantly different to the wild‐type (*P* > 0.05, Student's *T*‐test). The control CDΔtcdR_499_ did not appear to produce any TcdA above the limit of quantification (5 ng·mL^−1^), consistent with its role as a positive toxin regulator.

#### Adherence to the Caco‐2 colonic cell line

The ability of the cwp2^−^ mutant to adhere to Caco‐2 cells *in vitro* was assessed, taking nonspecific binding into account (Fig. 4B). This showed that mean adhesion of the mutant (0.073 ± 0.047 bacteria/cell (SEM)) was significantly lower than the wild‐type (0.51 ± 0.18) (*P* < 0.05, Student's *t*‐test), a decrease of 85.7 ± 14.0% indicating that knockout of *cwp2* decreases the ability of *C. difficile* to adhere to Caco‐2 cells *in vitro*.

#### Capacity of cwp2^−^ to produce CDI *in vivo*

The hamster model was used to assess the capacity of cwp2^−^ to cause CDI. Test groups were challenged by the orogastric administration of spores produced from either cwp2^−^ or wild‐type *C. difficile* 630ΔErm 48 h after administration of clindamycin. Both groups displayed typical symptoms of CDI (diarrhoea, weight loss, lethargy). Figure 4C shows combined survival plots for these. The data suggest that cwp2^−^ was capable of causing CDI in the hamster model at a similar rate of onset and severity as the wild‐type strain.

The numbers of viable *C. difficile* vegetative cells and spores associated with tissues from the caecum were determined at the time of sacrifice by monitoring bacterial viable counts using both luminal washes and homogenized tissue from animals infected with cwp2^−^ or wild‐type *C. difficile* 630 ΔErm. No statistically significant differences were found in luminal (WT = 4.23 ± 0.93 log_10_ CFU·mL^−1^, KO = 4.14 ± 0.89 log_10_ CFU·mL^−1^) or tissue (WT = 3.94 ± 0.85 log_10_ CFU·mL^−1^, KO = 4.14 ± 0.75 log_10_ CFU·mL^−1^) associated bacterial counts.

## Discussion

Cwp2 is constitutively expressed and found on the surface of *C. difficile* during normal growth 16, 20; it is also found in the *C. difficile* 630 spore coat 17. However, the gene is not found in certain strains 13, indicating that it may not be essential for cell growth or pathogenesis. A significant number of CDI patients raise antibodies to Cwp2, which may suggest that antibodies raised against the protein are not protective 19. The presence of Cwp2 on the cell surface during normal growth does, however, suggest that it is required for some cellular process(s).

The results presented here show that although knocking out *cwp2* does not have a negative effect on the growth of *C. difficile*, its ability to form spores or to cause CDI in the hamster model, it has a decreased capacity to adhere *in vitro* and results in increased TcdA release. The former of these results suggests Cwp2 may play a role in host cell adhesion. This reflects the results previously seen for Cwp66 12 and LMW SLP 27.

Culture supernatants of the *cwp2* mutant, contained significantly higher levels of TcdA than the wild‐type *C. difficile* 630ΔErm after 24 h. This will either be due to increased toxin expression, increased toxin release or a combination of the two. As Cwp2 is surface located, it is possible that removal of the protein from the S‐layer results in decreased cell integrity. The resultant higher TcdA level may occur as a result of an increased susceptibility of toxin to ‘leak’ from the cell e.g. the cells lyse more readily – the alternative mechanism of toxin release to the proposed release via TcdE 28 Alternatively, unexplored pathways which result in altered transcription factors associated with toxin expression may be involved.

**Figure 5:**
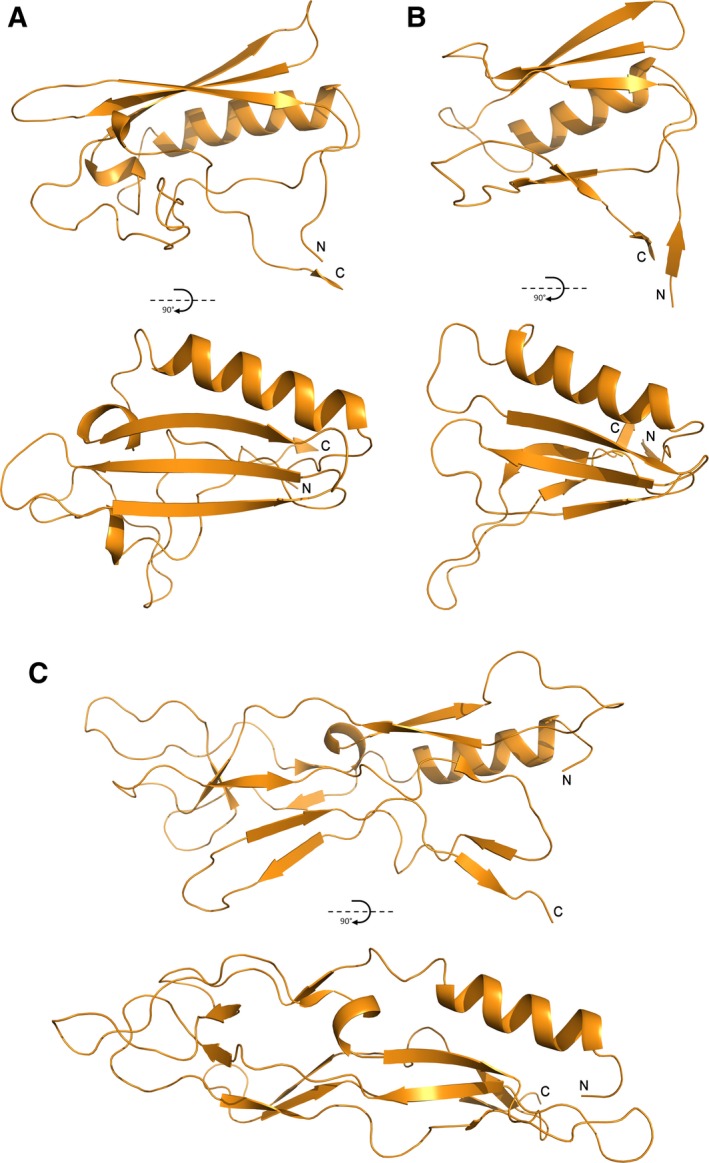
Comparison of Domain 2 of Cwp2, Cwp8 and LMW SLP – (A) Cwp2. (B) Cwp8. (C) LMW SLP. All three share a conserved α‐helix and β‐sheet. All three also possess extended loop regions whose structures are not conserved between the three proteins. The loop regions of LMW SLP, the variability of which has been linked to variations in adhesion, are considerably longer than those seen in Cwp2 and Cwp8.

In the recently reported structure of Cwp8, to which we have shown that Cwp2 bears significant structural similarity, Usenik *et al*. 24 demonstrated that domain 2 is likely to form the most surface exposed region of the protein, while domains 1 and 3 are more buried. This is also likely to be true for Cwp2 due to the high similarity between the two structures. Domain 2 in both proteins consists of an α‐helix, a three‐stranded β‐sheet and an extended loop region (Fig. 5), although there are significant differences in the loop regions. The two domains also show significantly different orientations in their respective structures, related to each other by a rotation of approximately 40° (Fig. 2). Domain 2 of LMW SLP, bears a level of similarity to domain 2 of Cwp2 and Cwp8. Although the β‐sheet in this protein has a slightly different orientation relative to the helix and the loop region is considerably larger.

Domain 2 of LMW SLP has been shown to exhibit low sequence identity between strains, which is likely to be permitted by the significant number of loops within the structure 22. The protein exhibits both immuno‐evasive capabilities 29 and adhesive properties 27. The variability of domain 2 has been suggested to play a role in immuno‐evasion 22 while variability of the whole protein has been shown to have an effect on the strength of cell adhesion 27. As domain 2 is very likely to be the most surface exposed part of the structure, it is possible that it is this domain that is primarily responsible for both immuno‐evasion and cell adhesion. Similarly, Cwp2 and Cwp8 both show increased amount of SNPs compared to other proteins within the slpA locus 13. It is therefore possible that variations in domain 2 of Cwp2 and Cwp8 may too have an effect on adhesive abilities and that the variation may be linked to immuno‐evasion. However, the cross‐reactivity of sera against Cwp2 15 casts doubt on the possibility of a role for Cwp2 in immuno‐evasion.

Despite the differences in orientation of domain 2 in Cwp2 and Cwp8 it is tempting to suggest that, the region between domains 1 and 2 may be able to act as a hinge allowing domain 2 to move relative to domains 1 and 3, so that it can assume an orientation closer to that observed for domain 2 in the structure of Cwp8 and vice versa. To examine this hypothesis, the flexibility of the two structures was computationally analysed using FIRST and FRODA. By probing the structures with physiologically relevant amounts of energy, it was possible to observe a hinging movement at the interface between domains 1 and 2 of Cwp2 and Cwp8 that allowed domain 2 to assume an orientation closer to that seen in the other structure (Fig. 2). In LMW SLP, the structure and orientation of domain 2 showed much greater differences than between Cwp2 and Cwp8, so it was not possible to observe the same orientation seen in the other two structures, however a hinging between the two domains was also seen.

**Figure 6:**
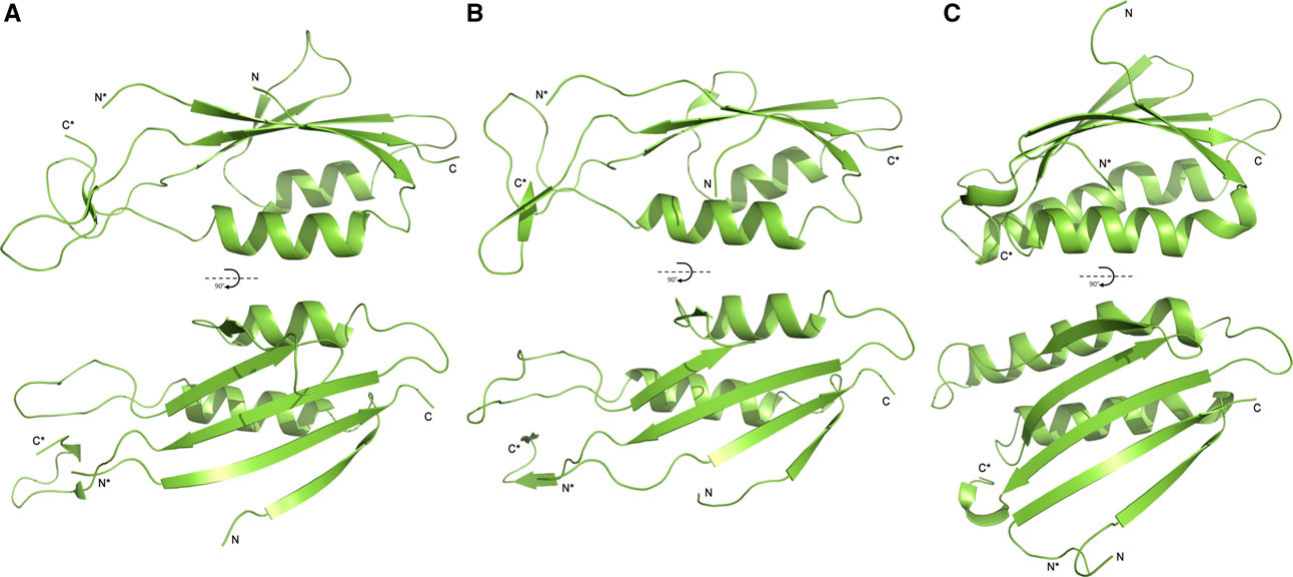
Comparison of Domain 1 of Cwp2, Cwp8 and LMW SLP – (A) Cwp2. (B) Cwp8. (C) LMW SLP. As domain 1 contains a single strand linking domains 2 and 3, the N‐ and C‐termini of the domain are labelled accordingly, while the portions connected to domain 2 are labelled C* and N*. The two‐layer sandwich structure of domain 1 is particularly well conserved between the three proteins. The only major differences are longer α‐helices in LMW SLP and different connections to domain 2, which shows significantly higher variation.

Domain 1 of Cwp2 possesses a very similar fold to that of domain 1 of LMW SLP and Cwp8 (Fig. 6) 22, 24. In all three proteins, the domain is likely to be more buried than domain 2. Domain 1 of LMW SLP shows a higher degree of conservation than domain 2, and is its structure considerably more conserved in Cwp2 and Cwp8 as well. It is therefore highly likely that they share a currently unidentified function.

**Figure 7:**
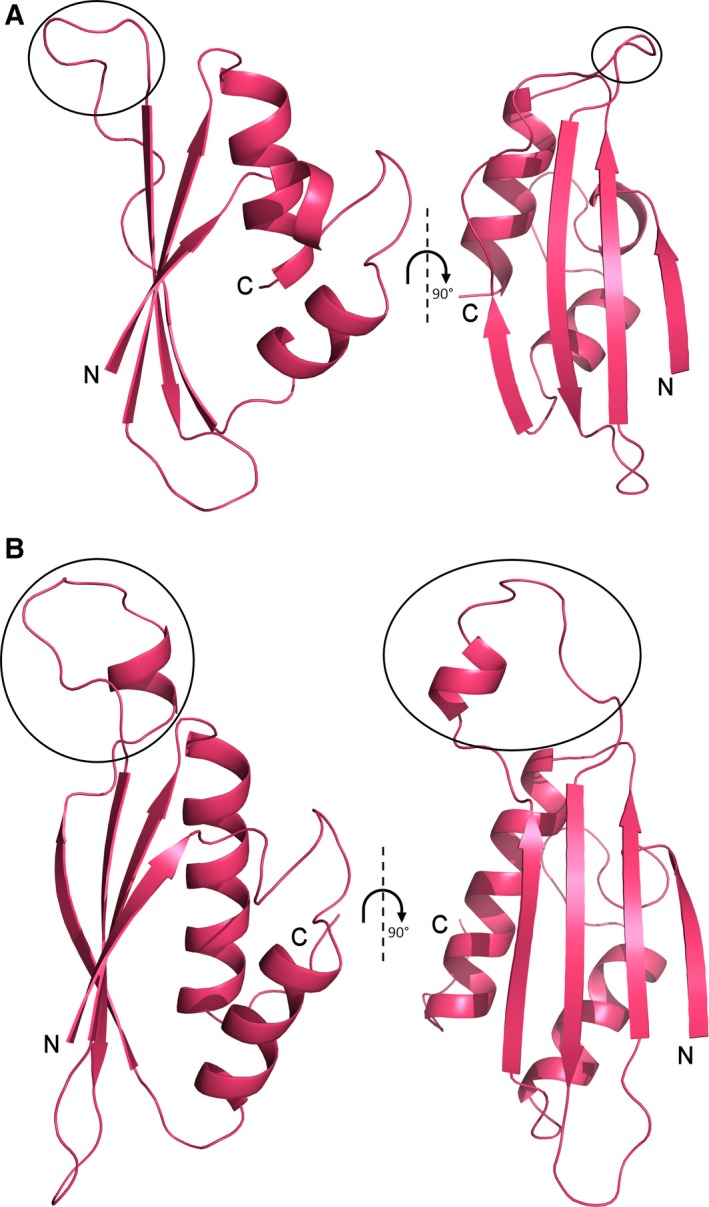
Comparison of Domain 3 of Cwp2, Cwp8 – (A) Cwp2. (B) Cwp8. In both proteins, domain 3 shows a similar two‐layer sandwich fold to domain 1. The most noticeable difference between the two is the loop region protruding from the top of the domain (circled), this region is much longer in Cwp8 than in Cwp2. In both structures, this region had poor density.

Domain 3 of Cwp2 and Cwp8 assumes a similar fold to domain 1 (Fig. 7), while the structure of domain 3 of LMW SLP, which has been shown to be responsible for the formation of the H/L complex 22, is currently unknown. The structure of Cwp8 demonstrates that there is a significant amount of interaction between domain 3 and the first cell wall‐binding domain (CWBD). It therefore seems logical that the fold of this domain will be optimized for interaction with the CWBDs. It may, therefore, be that domain 3 of LMW SLP bears some similarity to that of Cwp2 and Cwp8, although, in the case of Cwp2 and Cwp8, the attachment to the cell wall‐binding domains is covalent, while the interaction of LMW SLP to HMW SLP is noncovalent 22.

Usenik *et al*. noted three portions of the functional region of Cwp8 that showed poor electron density in their structure, notably, two of these are found in domain 2 and are likely to form the most surface exposed areas of the protein *in vivo*. The equivalent regions in Cwp2, Lys141‐Asp153 and Asn195‐Lys218 are much more ordered. The latter of these two regions is also considerably longer in Cwp2, assumes a significantly different conformation and is stabilized by crystal packing while the former is ordered despite being solvent exposed. The third region identified in Cwp8 was an exposed loop in domain 3 (Fig. 7). This loop also shows poor density in Cwp2 (Ile274‐Tyr284), but is much shorter than in Cwp8. Interestingly, these three regions show the lowest degree of sequence similarity between the two proteins 25.

This study has determined the structure of the ‘functional’ region of the *C. difficile* S‐layer‐associated protein Cwp2. The protein bears a high degree of similarity to the recently reported three‐dimensional structure of Cwp8 24, with an extended fold consisting of three domains. Domains 1 and 3 show a high degree of structural similarity, while domain 2 is separated by a hinge, shows the greatest degree of variation between the proteins and appears to be capable of assuming different orientations.

This study has also demonstrated that Cwp2 may exhibit adhesive properties similar to those exhibited by the related proteins, LMW SLP and Cwp66. Variations in the sequence of LMW SLP cause changes to the adhesive strength of the protein 27. The majority variation in LMW SLP is seen in domain 2 22, so it follows logically that this domain plays a primary role in adhesion, a feature that is likely to be shared by domain 2 of Cwp2 and Cwp8.

While an *in vitro* assay using Caco‐2 cells demonstrated significantly reduced adhesion of cwp2^−^ compared to the wild‐type control, the numbers of vegetative cells and spores adhering to hamster caecal tissue were not significantly different between the wild‐type and the mutant strain. This may reflect differences in the cell surface make‐up of human (Caco‐2) and hamster epithelial cells. Clearly, further detailed investigation is required to establish a role for Cwp2 in the process of *C. difficile* adhesion and colonization.

## Experimental procedures

### Crystallographic studies

#### Cloning, expression and purification

A synthetically synthesized gene encoding the ‘functional’ region of *cwp2* from *C. difficile* (residues 27–322 from strain QCD32g‐58; ribotype 027, Thermofisher, Carlsbad, CA, USA) was cloned by PCR into the SUMO tagged expression vector pET‐SUMO. The plasmid was transformed into *Escherichia coli* BL21*(DE3) cells, which were grown to an OD_600_ of 0.6 before expression was induced with 1 mm IPTG and continued overnight at 16 °C. Harvested cell pellets were lysed by sonication and His‐SUMO tagged proteins were first purified by Nickel IMAC. Eluted fractions were dialysed into SUMO protease cleavage buffer (50 mm tris‐HCl, 150 mm NaCl pH 8.0, 0.2% igepal, 1 mm DTT), then cleaved with SUMO protease (Thermofisher). A second round of IMAC was performed to remove any uncleaved material, with cleaved untagged Cwp2_27–322_ protein not binding. To remove residual detergent, the sample was dialysed into 20 mm Tris pH8.0 and purified on a Q‐sepharose column, eluted via a NaCl gradient. Following this, gel filtration using a Superdex 200 10/300 with PBS was undertaken. Eluted protein was dialysed into 20 mm Tris pH8.0 before concentration to 12.04 mg·mL^−1^ with a vivaspin 10 000 MWCO centrifugal concentrator prior to crystallization trial setup.

#### Crystallization, X‐ray diffraction data collection and processing

Crystallization‐condition screening was performed with a range of preprepared 96‐well screens (Molecular Dimensions) using an Art Robbins Phoenix nanodispensing robot. Crystals of Cwp2_27–322_ were observed in JCSG+ condition G11 (2.0 m ammonium sulphate, 0.1 m bis‐tris, pH 5.5) after a prolonged incubation period and were cryo‐protected by addition of ammonium sulphate to a final concentration of approximately 3 m before flash cooling in liquid nitrogen. Four datasets consisting of 900 images with 0.2° oscillations were collected from four crystals on beamline I04 at Diamond Light Source. Data were indexed and integrated separately with dials 30 and scaled together with aimless 31. Molecular replacement was performed with phaser 32 using three polyalanine models based on the three functional domains of Cwp8 (PDB: 5J6Q) 24. Domains 1, 2 and 3 yielded translation function Z‐scores of 8.26, 7.11 and 8.46 respectively, indicating that the solutions were likely to be correct, however the resulting electron density was still ambiguous. Refinement with refmac5 33 gave *R*_work_ and *R*_free_ scores of 0.519 and 0.528 respectively. Density modification was performed with parrot 34 and automated model building and refinement was performed with buccaneer 35 and refmac5. This resulted in *R*_work_ and *R*_free_ values of 0.469 and 0.506. The resulting model was optimized with manual building with COOT 36 and refinement with REFMAC5 to final *R*_work_ and *R*_free_ scores of 0.216 and 0.245. The structure was validated with molprobity 37. Crystallographic statistics are given in Table 1.

#### Flexibility simulation

The structures of Cwp2, Cwp8 24 and LMW SLP 22 were prepared for flexibility analysis using pymol (www.pymol.org) and molprobity. Normal mode eigenvectors were determined for the structures of the proteins using elnemo 38 while first 39 and froda 40 were used to determine flexibility of the proteins. Ten nontrivial modes were analysed and inspected with PyMol to identify modes of motion that resulted in greater similarities between the structures.

### Knockout studies

The ClosTron gene knockout system 41 was used for insertional inactivation of *cwp2* using protocols described previously 21. An Intron integration site within the gene was selected between positions 1569 and 1570. Intron re‐targeting IBS, EBS1d and EBS2 PCR primers were designed using the Sigma Aldrich TargeTron Design Site (Poole, UK). All primer sequences are given in Table 3.

The 353 bp region containing the IBS, EBS1d and EBS2 sequences responsible for intron specificity was synthetically synthesized based on *in silico* mutation of an intron re‐targeting region template using primers designed by the relevant intron re‐targeting site. This 353 bp region was then cloned into pMTL007C‐E2 via the HindIII and BsrGI sites and sequenced *in situ* with primers CspFdx‐F1 and pMTL007‐R1. The derivative pMTL007 plasmid construct(s) were transformed, via electroporation, into the conjugative donor *E. coli* CA434 and then transferred, via conjugation, into *C. difficile* 630ΔErm 42.

Successful transconjugants and subsequent integrants were selected for by streaking onto *C. difficile* selective media (E&O Labs, Bonnybridge, UK) with the addition of thiamphenicol (15 μg·mL^−1^) and then on sBHI agar containing erythromycin (5 μg·mL^−1^), respectively. Confirmation of knockout was performed using PCR with primers flanking the intron insertion site and using the ErmRAM primers. All *C. difficile* incubations were performed under anaerobic conditions.

#### RNA extraction

*Clostridium difficile* was re‐streaked on fastidious anaerobe agar (FAA) plates and incubated for 24 h at 37 °C. An appropriate culture volume was inoculated with a streak of colonies and grown overnight at 37 °C. Total RNA was extracted from the culture using RNeasy mini kit (Qiagen, Hilden, Germany) with enzymatic lysis and proteinase K digestion as per manufacturer's instructions. Total RNA was treated with Turbo DNAse™ (Ambion, Foster City, CA, USA).

#### Southern blot analysis

For Southern hybridization, gDNA was digested with HindIII, run on a 1.0% agarose gel, blotted to a positively charged nylon membrane, and hybridized with a DIG‐labelled probe corresponding to a 398 bp region at the end of the intron. The intron‐specific probe was generated by PCR using *cwp84* knockout gDNA 21 as a template and primers pMTL007_F1 and pMTL007‐R1. The process of Southern analysis was carried out using the DIG High Prime DNA Labeling and Detection Starter Kit I (Roche, Basel, Switzerland) according to the manufacturer's instructions.

#### Growth rate determination

Triplicate 20 mL sBHI broth was inoculated with three streaks of colonies and incubated for 16 h. The overnight culture was inoculated into fresh triplicate 20 mL sBHI broth such that the OD_600_ was approximately 0.1. At hourly intervals, the culture was agitated and samples removed and OD_600_ measured. OD_600_ was plotted against time and the linear part of the curve was used to determine a growth rate.

#### Sporulation

A stirred 80 mL sBHI culture was inoculated with a streak of colonies and incubated for 18 days. After this period, samples were taken and plated in dilution, pre and post heat shock (62 °C, 40 min), to give viable counts (colony forming units·mL^−1^).

#### Preparation of *Clostridium difficile* spores

A portion of the cwp2^−^ culture (300 μL) was spread onto FAA plates and incubated at 37 °C for 10 days in an anaerobic jar. After incubation, the bacterial lawn was scraped off and transferred to 15 mL of sterile Dulbecco's modified Eagle's medium (DMEM) and centrifuged for 20 min at 2000 ***g***. The resulting pellet was washed twice by re‐suspension into 12 mL DMEM followed by centrifugation and then re‐suspended into DMEM and heat shocked at 62 °C for 40 min. The resulting crude spore suspension was aliquoted and stored at −80 °C. Samples (200 μL) were used to obtain viable counts after heat shocking.

#### Toxin ELISA

Affinity purified sheep anti‐toxoid A and B IgGs were biotinylated using EZ‐Link^®^ Sulfo‐NHS‐Biotin reagents (Pierce, Rochford, IL, USA) as per manufacturer's instructions. Prior to and after biotinylation IgGs were dialysed against 50 mm HEPES, 0.15 m NaCl pH 7.4 overnight at 4 °C. Unbiotinylated sheep anti‐toxoid A and B IgGs were coated onto individual Maxisorb plates at 5 μg·mL^−1^ in PBS and incubated overnight at 4 °C. Plates were washed three times with PBS with 0.1% Tween 20 (Sigma, Poole, UK) (PBST) followed by application of PBST with 5% FBS (blocking buffer) and incubated for 1 h at 37 °C. Plates were washed a further three times before the application of appropriate samples and TcdA and TcdB standards (diluted to 1000 ng·mL^−1^) and incubated for 1 h at 37 °C. Plates were washed a further three times before application of either biotinylated sheep anti‐toxoid A (1 μg·mL^−1^) or B IgG (10 μg·mL^−1^) and incubated for 1 h at 37 °C. Plates were washed as before then streptavidin‐HRP conjugate (GE Healthcare, Chalfont St Giles, UK) (1 : 1000, in blocking buffer) applied and incubated for 10 min followed by a final three washes. Detection was undertaken with the addition of a TMB substrate and TMB stop solution (BioFX, Eden Prairie, MN, USA). The absorbance of each well was read at 450 nm using a Tecan™ Sunrise microtitre plate reader. Toxin concentrations were estimated using a sigmoidal four‐parameter logistic standard curve.

*Clostridium difficile* culture supernatants for TcdA or TcdB ELISA were obtained by removing bacterial cells from *C. difficile* cultures by centrifugation, then dialysing the culture supernatant for 24 h into 50 mm HEPES 0.15 m NaCl, pH 7.4, using 12 kDa MWCO dialysis tubing before storage at −80 °C if not used directly.

#### Surface protein extraction

Surface protein extraction was performed using the low pH glycine method 8, 43. Briefly, the harvested cell pellets were washed once with 10 mm HEPES, 100 mm NaCl, pH 7.4, re‐suspended in 0.2 m glycine, pH 2.2 and incubated for 30 min at 22 °C. Cells were then removed by centrifugation and the supernatant neutralized with 2 m Tris‐HCl, pH 9.0. Extracted SLPs were dialysed into 50 mm HEPES containing 0.15 m, NaCl pH 7.4 and stored at −80 °C if not used directly.

#### Adhesion assay

Caco‐2 cells were seeded into eight well Lab‐Tek^TM^ chamber slides at a 1 : 2 dilution in Minimum Essential Medium Eagle medium (MEME) (approx 5.69 × 10^4^ cells per well) for 14 days prior to the assay to ensure full differentiation. 18 h *C. difficile* culture was harvested by centrifugation, then washed twice with an equal volume of Dulbecco's phosphate buffered saline (DPBS), before re‐suspension in MEME ± ovine anti‐R20291 SLP IgG (in 50 mm HEPES 0.15 m NaCl pH 7.4) or ovine anti‐R20291 crude spore serum as a control against nonspecific binding. Caco‐2 cells in chamber slides were washed twice with 0.4 mL per well of DPBS before addition of 0.4 mL of bacterial suspension to each well. Slides were incubated for 2 h 20 min at 37 °C in anaerobic conditions followed by washing three times with DPBS. Slides were then fixed with methanol at −20 °C for 5–10 min and stained with Giemsa stain (Sigma) diluted with Wright‐Giemsa stain phosphate buffer pH 6.8 (Park Scientific Ltd, Northampton, UK) before microscopic observation at ×1000 magnification using an oil immersion lens. Enumeration was performed using a single‐blind experimental design. Each experiment was performed with 20 replicates, mean‐specific binding was calculated as binding without antiserum minus binding with antiserum.

#### Animal model for *Clostridium difficile* infection

Syrian hamsters were challenged with cwp2^−^ as previously described 21. Briefly, each hamster in two groups of ten hamsters was orgastrically administered 200 colony forming units of either *C. difficile* 630 wild‐type spores or cwp2^−^ spores in 0.2 mL DMEM 2 days after clindamycin administration, while eight control hamsters just received DMEM. All animals were monitored six times a day for 12 days for signs of diarrhoea, weight loss, lethargy and tender abdomen. Animals in advanced stages of disease which had become immobile were euthanized. Faecal samples were taken from euthanized animals by colectomy and *C. difficile* cultured as described above.

To determine pathology, the tip of the caecum was removed and placed into 10% neutral buffered formalin prior to histology by haematoxylin and eosin staining. To enumerate the total bacterial load (spores and vegetative cells), the rest of the caecum was opened longitudinally, and the contents were removed by gentle washing in two changes of 10 mL sterile PBS, 10% DMSO, then frozen at −80 °C until processing. The tissues were homogenized in 5 mL of PBS, 10% DMSO for 2 min using a stomacher, and viable were measured counts as described above.

The PCR was undertaken on selected colonies grown from cultures to ensure the strain isolated was the same strain as used in the challenge.

## Conflict of interest

The authors declare that they have no conflicts of interest with the contents of this article.

## Author contributions

WJB performed structural biology experiments, analysed the structures and wrote the manuscript. JMK performed protein expression, purification and structural biology experiments, all biological experiments, analysed the data and edited the manuscript. AKR supervised some of the work and edited the manuscript. KRA and CCS conceived and supervised the study, analysed the data and edited the manuscript. All authors reviewed the manuscript.

## References

[1] Barkin JA , Sussman DA , Fifadara N & Barkin JS (2017) *Clostridium difficile* infection and patient‐specific antimicrobial resistance testing reveals a high metronidazole resistance rate. Dig Dis Sci 62, 1035–1042. 28116592 10.1007/s10620-017-4462-9

[2] Ong GK , Reidy TJ , Huk MD & Lane FR (2017) *Clostridium difficile* colitis: a clinical review. Am J Surg 213, 565–571. 28131326 10.1016/j.amjsurg.2016.10.035

[3] Davies AH , Roberts AK , Shone CC & Acharya KR (2011) Super toxins from a super bug: structure and function of *Clostridium difficile* toxins. Biochem J 436, 517–526. 21615333 10.1042/BJ20110106

[4] Voth DE & Ballard JD (2005) *Clostridium difficile* toxins: mechanism of action and role in disease. Clin Microbiol Rev 18, 247–263. 15831824 10.1128/CMR.18.2.247-263.2005 PMC1082799

[5] Sara M & Sleytr UB (2000) S‐Layer proteins. J Bacteriol 182, 859–868. 10648507 10.1128/jb.182.4.859-868.2000 PMC94357

[6] Fagan RP & Fairweather NF (2014) Biogenesis and functions of bacterial S‐layers. Nat Rev Microbiol 12, 211–222. 24509785 10.1038/nrmicro3213

[7] Willing SE , Candela T , Shaw HA , Seager Z , Mesnage S , Fagan RP & Fairweather NF (2015) *Clostridium difficile* surface proteins are anchored to the cell wall using CWB2 motifs that recognise the anionic polymer PSII. Mol Microbiol 96, 596–608. 25649385 10.1111/mmi.12958 PMC4973711

[8] Calabi E , Ward S , Wren B , Paxton T , Panico M , Morris H , Dell A , Dougan G & Fairweather N (2001) Molecular characterization of the surface layer proteins from *Clostridium difficile* . Mol Microbiol 40, 1187–1199. 11401722 10.1046/j.1365-2958.2001.02461.x

[9] Karjalainen T , Waligora‐Dupriet AJ , Cerquetti M , Spigaglia P , Maggioni A , Mauri P & Mastrantonio P (2001) Molecular and genomic analysis of genes encoding surface‐anchored proteins from *Clostridium difficile* . Infect Immun 69, 3442–3446. 11292772 10.1128/IAI.69.5.3442-3446.2001 PMC98308

[10] Savariau‐Lacomme MP , Lebarbier C , Karjalainen T , Collignon A & Janoir C (2003) Transcription and analysis of polymorphism in a cluster of genes encoding surface‐associated proteins of *Clostridium difficile* . J Bacteriol 185, 4461–4470. 12867455 10.1128/JB.185.15.4461-4470.2003 PMC165755

[11] Baerends RJ , Smits WK , de Jong A , Hamoen LW , Kok J & Kuipers OP (2004) Genome2D: a visualization tool for the rapid analysis of bacterial transcriptome data. Genome Biol 5, R37. 15128451 10.1186/gb-2004-5-5-r37 PMC416473

[12] Waligora AJ , Hennequin C , Mullany P , Bourlioux P , Collignon A & Karjalainen T (2001) Characterization of a cell surface protein of *Clostridium difficile* with adhesive properties. Infect Immun 69, 2144–2153. 11254569 10.1128/IAI.69.4.2144-2153.2001 PMC98141

[13] Dingle KE , Didelot X , Ansari MA , Eyre DW , Vaughan A , Griffiths D , Ip CL , Batty EM , Golubchik T , Bowden R *et al* (2013) Recombinational switching of the *Clostridium difficile* S‐layer and a novel glycosylation gene cluster revealed by large‐scale whole‐genome sequencing. J Infect Dis 207, 675–686. 23204167 10.1093/infdis/jis734 PMC3549603

[14] Emerson JE , Stabler RA , Wren BW & Fairweather NF (2008) Microarray analysis of the transcriptional responses of *Clostridium difficile* to environmental and antibiotic stress. J Med Microbiol 57, 757–764. 18480334 10.1099/jmm.0.47657-0

[15] McCoubrey J & Poxton IR (2001) Variation in the surface layer proteins of *Clostridium difficile* . FEMS Immunol Med Microbiol 31, 131–135. 11549420 10.1111/j.1574-695X.2001.tb00509.x

[16] Wright A , Wait R , Begum S , Crossett B , Nagy J , Brown K & Fairweather N (2005) Proteomic analysis of cell surface proteins from *Clostridium difficile* . Proteomics 5, 2443–2452. 15887182 10.1002/pmic.200401179

[17] Lawley TD , Croucher NJ , Yu L , Clare S , Sebaihia M , Goulding D , Pickard DJ , Parkhill J , Choudhary J & Dougan G (2009) Proteomic and genomic characterization of highly infectious *Clostridium difficile* 630 spores. J Bacteriol 191, 5377–5386. 19542279 10.1128/JB.00597-09 PMC2725610

[18] Mukherjee K , Karlsson S , Burman LG & Akerlund T (2002) Proteins released during high toxin production in *Clostridium difficile* . Microbiology 148, 2245–2453. 12101311 10.1099/00221287-148-7-2245

[19] Wright A , Drudy D , Kyne L , Brown K & Fairweather NF (2008) Immunoreactive cell wall proteins of *Clostridium difficile* identified by human sera. J Med Microbiol 57, 750–756. 18480333 10.1099/jmm.0.47532-0

[20] Calabi E & Fairweather N (2002) Patterns of sequence conservation in the S‐Layer proteins and related sequences in *Clostridium difficile* . J Bacteriol 184, 3886–3897. 12081960 10.1128/JB.184.14.3886-3897.2002 PMC135169

[21] Kirby JM , Ahern H , Roberts AK , Kumar V , Freeman Z , Acharya KR & Shone CC (2009) Cwp84, a surface‐associated cysteine protease, plays a role in the maturation of the surface layer of *Clostridium difficile* . J Biol Chem 284, 34666–34673. 19808679 10.1074/jbc.M109.051177 PMC2787329

[22] Fagan RP , Albesa‐Jove D , Qazi O , Svergun DI , Brown KA & Fairweather NF (2009) Structural insights into the molecular organization of the S‐layer from *Clostridium difficile* . Mol Microbiol 71, 1308–1322. 19183279 10.1111/j.1365-2958.2009.06603.x

[23] Bradshaw WJ , Kirby JM , Thiyagarajan N , Chambers CJ , Davies AH , Roberts AK , Shone CC & Acharya KR (2014) The structure of the cysteine protease and lectin‐like domains of Cwp84, a surface layer‐associated protein from *Clostridium difficile* . Acta Crystallogr D Biol Crystallogr 70, 1983–1993. 25004975 10.1107/S1399004714009997 PMC4089489

[24] Usenik A , Renko M , Mihelic M , Lindic N , Borisek J , Perdih A , Pretnar G , Muller U & Turk D (2017) The CWB2 cell wall‐anchoring module is revealed by the crystal structures of the *Clostridium difficile* cell wall proteins Cwp8 and Cwp6. Structure 7, 514–521. 10.1016/j.str.2016.12.018 28132783

[25] Altschul SF , Gish W , Miller W , Myers EW & Lipman DJ (1990) Basic local alignment search tool. J Mol Biol 215, 403–410. 2231712 10.1016/S0022-2836(05)80360-2

[26] Merrigan M , Venugopal A , Mallozzi M , Roxas B , Viswanathan VK , Johnson S , Gerding DN & Vedantam G (2010) Human hypervirulent *Clostridium difficile* strains exhibit increased sporulation as well as robust toxin production. J Bacteriol 192, 4904–4911. 20675495 10.1128/JB.00445-10 PMC2944552

[27] Merrigan MM , Venugopal A , Roxas JL , Anwar F , Mallozzi MJ , Roxas BA , Gerding DN , Viswanathan VK & Vedantam G (2013) Surface‐layer protein A (SlpA) is a major contributor to host‐cell adherence of *Clostridium difficile* . PLoS One 8, e78404. 24265687 10.1371/journal.pone.0078404 PMC3827033

[28] Tan KS , Wee BY & Song KP (2001) Evidence for holin function of tcdE gene in the pathogenicity of *Clostridium difficile* . J Med Microbiol 50, 613–619. 11444771 10.1099/0022-1317-50-7-613

[29] Spigaglia P , Galeotti CL , Barbanti F , Scarselli M , Van Broeck J & Mastrantonio P (2011) The LMW surface‐layer proteins of *Clostridium difficile* PCR ribotypes 027 and 001 share common immunogenic properties. J Med Microbiol 60, 1168–1173. 21349988 10.1099/jmm.0.029710-0

[30] Waterman DG , Winter G , Gildea RJ , Parkhurst JM , Brewster AS , Sauter NK & Evans G (2016) Diffraction‐geometry refinement in the DIALS framework. Acta Crystallogr D Struct Biol 72, 558–575. 27050135 10.1107/S2059798316002187 PMC4822564

[31] Evans PR & Murshudov GN (2013) How good are my data and what is the resolution? Acta Crystallogr D Biol Crystallogr 69, 1204–1214. 23793146 10.1107/S0907444913000061 PMC3689523

[32] McCoy AJ , Grosse‐Kunstleve RW , Adams PD , Winn MD , Storoni LC & Read RJ (2007) Phaser crystallographic software. J Appl Crystallogr 40, 658–674. 19461840 10.1107/S0021889807021206 PMC2483472

[33] Murshudov GN , Skubak P , Lebedev AA , Pannu NS , Steiner RA , Nicholls RA , Winn MD , Long F & Vagin AA (2011) REFMAC5 for the refinement of macromolecular crystal structures. Acta Crystallogr D Biol Crystallogr 67, 355–367. 21460454 10.1107/S0907444911001314 PMC3069751

[34] Cowtan K (2010) Recent developments in classical density modification. Acta Crystallogr D Biol Crystallogr 66, 470–478. 20383000 10.1107/S090744490903947X PMC2852311

[35] Cowtan K (2006) The Buccaneer software for automated model building. 1. Tracing protein chains. Acta Crystallogr D Biol Crystallogr 62, 1002–1011. 16929101 10.1107/S0907444906022116

[36] Emsley P & Cowtan K (2004) Coot: model‐building tools for molecular graphics. Acta Crystallogr D Biol Crystallogr 60, 2126–2132. 15572765 10.1107/S0907444904019158

[37] Chen VB , Arendall WB 3rd , Headd JJ , Keedy DA , Immormino RM , Kapral GJ , Murray LW , Richardson JS & Richardson DC (2010) MolProbity: all‐atom structure validation for macromolecular crystallography. Acta Crystallogr D Biol Crystallogr 66, 12–21. 20057044 10.1107/S0907444909042073 PMC2803126

[38] Suhre K & Sanejouand YH (2004) ElNemo: a normal mode web server for protein movement analysis and the generation of templates for molecular replacement. Nucleic Acids Res 32, W610–W614. 15215461 10.1093/nar/gkh368 PMC441506

[39] Jacobs DJ , Rader AJ , Kuhn LA & Thorpe MF (2001) Protein flexibility predictions using graph theory. Proteins 44, 150–165. 11391777 10.1002/prot.1081

[40] Wells S , Menor S , Hespenheide B & Thorpe MF (2005) Constrained geometric simulation of diffusive motion in proteins. Phys Biol 2, S127–S136. 16280618 10.1088/1478-3975/2/4/S07

[41] Heap JT , Pennington OJ , Cartman ST , Carter GP & Minton NP (2007) The ClosTron: a universal gene knock‐out system for the genus *Clostridium* . J Microbiol Methods 70, 452–464. 17658189 10.1016/j.mimet.2007.05.021

[42] Purdy D , O'Keeffe TA , Elmore M , Herbert M , McLeod A , Bokori‐Brown M , Ostrowski A & Minton NP (2002) Conjugative transfer of clostridial shuttle vectors from *Escherichia coli* to *Clostridium difficile* through circumvention of the restriction barrier. Mol Microbiol 46, 439–452. 12406220 10.1046/j.1365-2958.2002.03134.x

[43] Cerquetti M , Molinari A , Sebastianelli A , Diociaiuti M , Petruzzelli R , Capo C & Mastrantonio P (2000) Characterization of surface layer proteins from different *Clostridium difficile* clinical isolates. Microb Pathog 28, 363–372. 10839973 10.1006/mpat.2000.0356

